# Women’s Politics of Solidarity in El Salvador: Familial Love, Carceral Peace, and Patriarchy

**DOI:** 10.1007/s10612-025-09855-y

**Published:** 2025-09-29

**Authors:** R. Elizabeth Velásquez Estrada

**Affiliations:** University of Illinois Urbana-Champaign, 1207 West Oregon Street, Urbana, IL 61801 USA

## Abstract

El Salvador’s U.S.-inspired war on gangs and mass incarceration is lauded by many globally as the needed punitive solution to end gang violence. However, critical gang studies challenge this view, emphasizing gangs’ embeddedness within social, economic, and political systems that shape and sustain their violence. In this oral history, I expand critical gang studies’ discussion on relationality by incorporating a gendered analysis of interdependence related to familial love. From 2022 to 2024, I documented the life history of Arquímedes, a prominent *Mara Salvatrucha* (MS-13) leader in El Salvador, through the perspective of and interviews with his cousin, Alicia. I argue that women relatives of male gang members’ complex practices of solidarity reveal how patriarchy shapes dynamics across scales, fueling gang and state violence as a practice of protection for family and nation. Failing to address patriarchy condemns security efforts to ongoing violence as part of state measures to attain peace.

## Introduction

In 2022 I returned to San Salvador to carry out research for a global ethnography project on gangs^1^ and to document the life trajectory of Arquímedes,^2^ a *Mara Salvatrucha* (MS-13) gang leader, as told from the perspective of his cousin, Alicia. She had been a support and advocate for him through his many years of incarceration and was excited to share Arquímedes’ life trajectory with me. She wanted people to know who Arquímedes was as a person beyond the depictions of him as a terrifying MS-13 gang leader. Yet as our conversations deepened over two years, it became clear that this was Alicia’s story, too. Documenting Arquímedes’ life trajectory from Alicia’s perspective reveals how their lives are intertwined through family intimacy and the values of care and protection that operate across their communities. The relationship between Alicia and Arquímedes reveals that male gang members’ life trajectories move beyond individual biography, asking us to develop an understanding of their interdependent relationships. This oral history traces how deeply embedded gender expectations shape intimate family relationships and women’s politics of solidarity within gang life in El Salvador, highlighting how they simultaneously support and critique their male relatives’ practices of resistance and violence. My conversations with Alicia also provide us with a path to understand how women’s socially constituted and gendered duty of care is being transformed, politicized, and reaffirmed under conditions of a punitive security state.

The phenomenon of gangs in El Salvador, particularly MS-13 and *Barrio 18* (18th Street), has attracted considerable local and international scholarly attention (Bruneau et al., 2011; Cruz, 2014). After the signing of the 1992 Peace Accords ended El Salvador’s 12-year civil war, the U.S. government increased the number of Salvadorans they deported, including gang members. In the post-Accords era, homicides and femicides made the country one of the world’s most violent (Moodie, 2010; Watts, 2015). The Salvadoran state held gangs responsible for this violence, labeling them a “plague,” adopting US-inspired punitive security policies, and designating them as terrorist groups (Wolf, 2017; Zilberg, 2011). In 2012 the rival gangs, MS-13 and *Barrio 18*, negotiated a historic truce with each other and with the state that reduced daily homicides from 14 to 5, even while El Salvador still held the world’s highest femicide rate (Ayala, 2013; Nowak, 2012). The truce held until 2014 when the state abandoned it. Since then, the state has further normalized punitive security policies and the suppression of human rights, effectively creating what I call a "carceral peace," in which political control and peaceful communities presuppose punitive forms of justice. In addition to deploying the military alongside the police under a denominated “regime of exception,” ostensibly to reduce gang violence, anyone suspected of gang membership can be arrested, charged with terrorism, and jailed indefinitely (Amnesty International, 2024a, 2024b). The state is sanctioning human rights violations within the criminal justice system including violations of due process, enforced disappearances, torture, and murder, all justified as necessary measures to eradicate the gangs. The state justifies these policies by constructing gang members as a nonhuman “plague,” outside the imagined community of the Salvadoran nation, and therefore undeserving of rights.

A central debate in gang studies concerns the extent to which gangs are separated from or integrated into their communities. Much of the scholarship examines gangs as isolated, autonomous entities separate from their broader social environment. It is frequently argued that gangs are deviant, often criminal, subcultures with their own norms, values, and behaviors (Hirschi, 2002; Melossi, 2008). However, critical gang scholarship raises sharp critiques of this perspective, arguing that such a portrayal justifies repressive interventions by state and non-state actors, as these conditions are presumed to solely impact gangs, not the communities in which they are embedded (Brotherton, 2015; Hallsworth & Young, 2008; Kontos et al., 2003). Critical gang studies have highlighted the fundamentally relational character of gangs, stressing that gang members’ behavior, group perspectives, and identities are intertwined with, and mutually constitutive, of their relationships with family members, communities, and institutions that transcend the gang (Hagedorn, 2008; Jensen & Rodgers, 2022; Pyrooz et al., 2024, pp. 54–73). Within this literature, several scholars have theorized the role that patriarchy and dominant notions of masculinity have on gender dynamics within gangs’ intimacies and violence (Baird, 2021; Hume, 2008, 2009; IUDOP, 2010; Rodgers, 2024).

In this article, I contribute to scholarship on gang relationality by examining how a patriarchal logic of protection produces a politics of care that shapes gang interdependence. Feminist scholars urge us to examine how the patriarchal logic of the benevolent masculine protector operates across scales and justifies aggressive interventions as “politics of care” (Das, 2007; Young, 2003). This logic extends from the head of the family to national leaders, who position themselves as protectors of the family or national population to justify their interventions. It engenders external and internal “security regimes” and “human security governance” as a politics of care that treats populations as “victims” requiring saving (Abu-Lughod, 2002; Amar, 2013; Mohanty, 1984; Ticktin, 2011; Young, 2003). The patriarchal politics of care overlooks the power asymmetries and inequality shaping social problems, frequently exacerbating them through punitive measures such as mass incarceration (Crenshaw, 2012; Hinton, 2016). Women relatives of the incarcerated find themselves challenging both the state’s and their relatives’ practices of violence even while supporting their incarcerated relatives (Aretxaga, 1997; Gilmore, 2022; Naber et al., 2020; Shirlow & Dowler, 2010).

Rooted in familial love, women in El Salvador have a long history of practicing solidarity and resisting punitive regimes and human rights violations. Whether joining human rights organizations or the FMLN (*Frente Farabundo Martí para la Liberación Nacional*, or Farabundo Marti National Liberation Front) guerrilla army, many women became politicized through their caregiving roles, risking their lives to confront state violence, including kidnapping, torture, rape, imprisonment, and disappearance (Cosgrove, 1999; Silber, 2011; Stephen, 1997; Viterna, 2013). Presently, an emergent women’s activism focuses on intersectional demands for justice as women are forced to care for their criminalized and often incarcerated relatives (Velásquez Estrada 2022: 11), while at the same time economically sustaining the very authoritarian regime that imprisons them (Grimaldi Calderon, 2024).

In this essay, I bring critical gang studies and feminist scholarship into conversation to examine (1) how women relatives of gang members perceive the patriarchal precept of protection and the gendered expectations that shape their family relationships and their relatives’ behavior as social actors; and (2) how what I have termed women’s “complex politics of solidarity” are leading to significant shifts in their awareness and practices as they respond to the assault on their communities under El Salvador’s current punitive security regime (Velásquez Estrada, 2026). I draw on intersectionality as theorized by feminist scholar Patricia Hill Collins (2019: 279) as an analytical tool to explore these issues. She urges us to move beyond “the independent/dependent binary notion” and center interdependence in the application of an intersectional analytical lens, contending that this helps bring to light “the interconnected, dynamic nature of intersecting power relations of the communities that organize such relations, and of relationships among individuals” that shape demands for social justice. Applying this to the study of gangs in El Salvador, I argue that a focus solely on gang members’ experiences risks neglecting the interconnection of familial love, punitive security policies, and patriarchal gendered expectations that shape gang relationality. More specifically, it risks overlooking the unique agency and labor of female relatives of gang members. Alicia’s perspective exposes the profound influence of deeply embedded gender expectations on both gang dynamics and family relationships. By tracing the interdependence between a gendered notion of protection, intimate family bonds, and advocacy within the context of Salvadoran gang life, this article offers a critical, gendered understanding of the complex relational landscape of gangs.

## Patriarchal Protection as a Security Policy

Post-Accords El Salvador has been shaped by neoliberal policies that have resulted in widespread inequality and pauperization. A generation of youth, ex-combatants, and deportees found themselves among the ranks of the economically disenfranchised. A number of deportees formed gangs that engaged in violence, territorial disputes, and extortion (*renta*) for survival. Because this emergent gang phenomenon, rather than the underlying economic issues, has been politicized, ending gang violence is widely projected as *the* key to achieving social peace. In 2003, citing violence against women, the right-wing ARENA (*Alianza Republicana Nacionalista*, or Nationalist Republican Alliance) government led by President Francisco Flores implemented the first zero-tolerance security policy to protect the nation against the “barbaric” violence of gangs (Hume, 2007). This policy, which they called “*Mano Dura*” (Iron Fist), criminalized gang membership based on superficial evidence, like tattoos and clothes. In 2009 the leftist FMLN government led by Mauricio Funes further linked violence against women to gangs, stating, “the cruel [gang] phenomenon we want to banish is born in violence against women… whoever attacks a woman assaults the nation…”.

Regardless of their political affiliation, successive Salvadoran governments have all invoked a particular narrative that draws on what I have termed elsewhere a “racist warring masculinity,” a gendered practice that legitimizes violence in order to demonstrate strength, resolve conflict, and protect an imagined (and racialized) community of the Salvadoran nation against those they consider purgeable non-human internal enemies (Velásquez Estrada, 2022). The overarching logic of racist warring masculinity represents the state as a “protector” of vulnerable populations, in particular, women and children, who need “saving.” It sees the use of, or capacity for, violence, specifically against women, as a measure of gangsters’ inhumanity, constructing gangs as a “savage/Other” internal enemy. It allows the state to distinguish between those who deserve justice and human rights protections, and those Others who must be combated and eliminated to attain the state’s punitive notion of justice. At the same time it actively promotes a gendered hierarchy. The state’s “protector” role can thus be said to constitute a patriarchal grammar of power that gang members embody when they position themselves as “protectors” of their communities against perceived threats – implicitly emanating from the state and non-state actors – and as they represent women as dependent and without agency, subordinated to male governance and protection.

It is within this context that I learned about Arquímedes through one of my research interlocutors, Diego, a former FMLN guerrilla combatant. Arquímedes is a poet and a gang leader who played a key role in the gang truce negotiations with the Salvadoran state. Born in the 1970s, he spent a prolonged period of incarceration within the Salvadoran prison system from the mid-1990s through to the late 2000s. While incarcerated, Arquímedes began writing poetry and became a human rights activist. He co-founded a prisoner-run organization dedicated to improving prison conditions and worked to expand education programs, building a classroom, and developing library resources. He denounced human rights violations in the prisons, such as lack of due process, prison guard brutality, and extrajudicial killings. Upon his release, he continued his advocacy work. However, in 2016, he was re-arrested for gang-related activities and has remained incarcerated ever since.

I only met Arquímedes once, when Diego organized an interview with him and two other gang members at a local burger joint near the MetroCentro mall in San Salvador. Arquímedes was wearing a long-sleeved dress shirt and glasses. His demeanor reminded me of a university student; he listened intently while letting the other gang members reply to my queries about their motivations for engaging in the truce. One gang member reiterated something I had heard from other male gang members: “I want to give my children a better life than I had.” This aspiration to protect his children highlighted for me how familial relationships can motivate gang members’ peacemaking. It also prompted me to reflect on how gang members’ deeply gendered perceptions of men as “protectors” influenced their relationships, not just with their families but also with the state.

When I met Arquímedes, my research had become bifurcated. While I found it crucial to understand gang members’ motivations and perspectives for engaging in grassroots peacemaking with rival gangs and the state, I also observed that women interlocutors’ voices and insights were overlooked in conversations about peacemaking. Women relatives of gang members were involved in continual negotiations to support their relatives while at the same time critiquing their violence and what I have termed elsewhere “masculinist peacemaking practices” (Velasquez Estrada, 2022). One finding of my ongoing research in El Salvador is that the patriarchal principle of protection, which guides not only key elements of the state’s punitive security measures but also gangs’ grassroots peacemaking, has had a profound impact on gangs’ relationality, shaping their relationships with the state and their families. But in contrast to the state and male gang members’ portrayal of women as vulnerable and in need of saving, women relatives of male gang members are exercising their agency by engaging in complex practices of solidarity. Women are transforming their roles as grandmothers, mothers, sisters, and cousins of gang members, and in the process politicizing everyday practices of care and reshaping how gangs, peace, and living are understood.

After my meeting with Arquímedes, Diego reconnected me with Arquímedes’ cousin, my former law school classmate, Alicia. I tried to meet with her, but it was a very difficult time. The Salvadoran government had entirely abandoned the truce negotiations. To pressure the state to return to negotiations, gangs were implementing curfews and national transportation strikes, threatening and killing bus drivers who dared to provide services. The Salvadoran government responded by increasing its punitive security measures, designating gangs as terrorist organizations, and creating a specialized tribunal to expedite cases involving gang members. Living conditions in gang-controlled neighborhoods resembled localized wars. Distrust and fear permeated the environment, and gang-controlled territories were almost hermetically sealed, making access difficult. My conversations with Alicia were postponed for “sometime later.”

## Gang Relationality: An Interdependent Approach

Although my acquaintance with Alicia was limited, I immediately liked her when we met in 1998 as first-year law students at the University of El Salvador, the only public university in the country. Alicia was one of the most committed and passionate students in the class, consistently striving to obtain the highest grades. She was reserved and seldom went out with at night. instead, she seemed to spend all her time studying with a small group of friends who had graduated from one of the city’s most prestigious private Jesuit schools. In the decade following the signing of the Peace Accords, a sense of newfound freedom permeated El Salvador. Many of us embraced the possibilities of peaceful living, including venturing out at night without fear of armed conflict. But not Alicia: her decision to forgo nightlife and dedicate herself to her education stood in stark contrast to the prevailing atmosphere in which most of my classmates and I participated.

This portrait of a hardworking Alicia accompanied me for decades. Every time I returned to El Salvador between 2001 and 2011, it was reinforced by updates from friends we had in common. I heard from them that Alicia graduated from law school and was studying to pass her notary exam.^3^ She had become a single mother, and when she confronted difficulties finding work, she opened a *tienda* (mini-store) in her home. All of this highlighted her resilience amid the social pressures that so many women encounter in El Salvador.

My eyes had blinked when I heard from Diego that Alicia was related to a gang member. Later, I was shocked to learn through my interviews with Alicia about the profound impact this relationship had on her life: it transformed her dream of becoming a poet into the goal of becoming a lawyer to advocate for justice for Arquímedes. It shaped in her a new understanding of familial love and human rights and committed her to life-long advocacy for criminalized relatives. Despite this labor of family love, Alicia, like many Salvadoran women, remained subordinated by her gender and by the pressure to navigate rigid patriarchal expectations. Salvadoran society, like many others, operates within a patriarchal framework that reinforces dominant gender roles. This framework assigns primary responsibility for domestic labor to women, including childcare, household upkeep, and catering to the needs of men. Conversely, men are expected to take on the roles of economic providers, protectors, and authority figures within the family structure and society. My research has shown that women relatives of gang members extend their caregiving role to advocate for justice for their relatives, even as they remain in a subordinated position due to their gender. Additionally, they are stigmatized and marginalized by both the state and broader society because of their kinship to gang members.

Although not members of the gangs, women relatives of gang members are often on the front lines, dealing with the consequences of gang violence, caring for their incarcerated family members, and navigating the social stigma and state criminalization associated with gang affiliation. As I came to understand this, I began to ask the following questions: How do women relatives of male gang members navigate the complex interplay of power relations, family love, and advocacy amidst systemic patriarchy and punitive security measures? What does their agency tell us about gangs’ relationship to patriarchy and its impacts? To answer these questions I draw on feminist political philosopher Joy James’ (2023: 16) concept of the “captive maternal,” understood as system-impacted women who engage in a “dialectical spiral of struggle moves from caretakers to protesters, into movement makers, marronage and forms of war resistance.” Salvadoran women are engaging with forms of care that move dialectically from doing care work to directly confronting the state’s notion of rights grounded in non-criminality, racialized humanity, citizenship, and belonging. Their resistance to the Salvadoran state’s war on gangs positions them as enemies of the nation who have no political rights or political material support. Nevertheless, with little time and energy and limited family economies, they continue their care work, even to the detriment of their health. In the process, they are reclaiming their affective freedom and beginning to liberate themselves from the gendered moral responsibilities that the Salvadoran state has imposed on them. Instead, they hold the state responsible for constructing the figures of the criminal and criminality, and for creating the political and economic conditions in which the phenomenon of the gangs has emerged.

The next section explores these issues through Alicia and Arquímedes’ relationship, which I was able to trace through a series of interviews that I carried out with Alicia between 2022 and 2024.

## You Will Be a Lawyer, and I Will Be a Poet

Alicia and Arquímedes have shared a close bond since childhood, growing up together in a working-class neighborhood in San Salvador. Alicia and her cousins had a joyful childhood, although it lacked luxuries. They had a place to call home, food, and toys. Alicia remembers Arquímedes as “a polite, respectful, and attentive child who always looked out for me,” and who acted as a caring mediator among her cousins despite being only five years older than her. Alicia highlights one particular memory of Arquímedes’ mediating role: “I had an orange tricycle that I never paid much attention to, but when Arquímedes and his brother Mario arrived, my cousin Mario and I fought over the tricycle. So Arquímedes told us, ‘I’m going to take care of you, but take turns’.” In many ways, Arquímedes was a model of good character and admirable behavior for Alicia, acting as her protector and using his influence to prevent conflicts among the cousins.

Alicia’s parents and grandparents shared her good opinion of him. Even as a child, he showed consideration for his single mother and relatives. Alicia remembers that Arquímedes’ mother, Maria, was a devout Assembly of God church member who supported her family by selling *pupusas* (thick, handmade corn tortillas filled with cheese, beans, pork, or a mix of these), and Arquímedes helped his mother with the *pupuseria* (pupusa restaurant). When Arquímedes and his brother visited Alicia’s home, Arquímedes consistently cleared the table and washed the dishes. “His actions impressed my mother, who would tell me, ‘Look at your cousin. He is very attentive’.”

From a young age, Alicia prized Arquímedes’ sharp intellect, which was nurtured by the father figures in his life. “During my father Felipe’s lifetime,” Alicia recalled, “Arquímedes was especially fond of talking with my dad. My dad loved to read. He taught a 10-year-old Arquímedes how to do acrostics. They would look for meaning in the letters that make up a word, making a sentence with each of them. Arquímedes loved to do acrostics.” Alicia recalled that Arquímedes treated her like a peer and confidant, telling her about his girlfriends and showing her the letters he wrote to them. As Alice and Arquímedes grew up they shared a passion for words. Alicia said, “Whenever we talked about our dreams, he said, ‘I will be a lawyer’, and I replied, ‘I will be a poet’.”

As a teenager, Arquímedes became a skilled and enthusiastic mechanic. Alicia recounted that at 16, he began working as a mechanic’s assistant in his uncle Ulises’s shop, which was always busy with a steady stream of customers. Uncle Ulises gave Arquímedes an opportunity to learn a trade while financially supporting his family. Alicia approved of Arquímedes learning a trade, but she believes that the ex-military men and other male adults who worked in the shop were a bad influence who taught him to drink. Nevertheless, with the support of relatives who provided opportunities his single mother could not offer, Arquímedes’ life seemed to be progressing.

A pivotal moment in the lives of Alicia and her family was Arquímedes’ arrest at age 20 on murder charges. “One morning my family and I woke up to the news that the police had arrested Arquímedes,” Alicia recalled. “We wondered, ‘what happened?’ […] For me, my cousin could be a *parrandero* (partygoer) or whatever, but not a murderer! …I started visiting him when he was in the Quezalte prison. To visit him I had to get my minority ID card, since I was 15 years old. I went with his brother, Mario.”^4^ Alicia remembered that “Arquímedes assured us of his innocence.” This was her family’s first exposure to the security system the state was putting in place to deal with gangs. They believed that the charges were invalid and were indignant that Arquímedes was held for two years without a trial.

As a teenager, Alicia provided Arquímedes with unwavering emotional support. While he was awaiting trial in prison, his mother died after a long illness. Alicia remembers delivering the heartbreaking news of his mother’s death during one of her regular visits. Arquímedes’ mother had been his unconditional support before she died. In the midst of her agony, she made Alicia’s mother promise to take care of him. Arquímedes had requested permission to visit his mother, but he was either denied or unable to arrive on time. The news of her death, compounded with his inability to visit her during her final days, was a tremendous emotional blow. Alicia remembers that Arquímedes took refuge in writing poems which he would share with her during her visits. Witnessing her cousin’s pain, Alicia grew more critical of the prison system.

During one of Alicia’s final visits to Arquímedes in Quezalte, Alicia made the crucial promise that would change her life. Alicia told Arquímedes, “I will study Law, the career you wanted. I promise to get you out of prison. When I graduate, I will demonstrate your innocence.” When I asked what motivated her, she replied, “Arquímedes was the brother I never had, and I was the sister he never had… He has protected me and trusted me since I was a child. When playing, he was the mediator and took care of us…” Alicia wishfully added, “Look at how things have turned out. I am now a lawyer, and he is a poet.”

But after making her life-changing promise, Alicia stopped visiting Arquímedes. Arquímedes had asked her not to visit because a guard made the daughter of another prisoner bleed while searching her body for items considered illegal to enter the prison with. Instead, Arquímedes began to make weekly calls. Alicia remembered, “During these calls, he would ask about my mother and my well-being.” Arquímedes told her about his and fellow prisoners’ efforts to improve prison conditions, including arranging for more frequent visits, intimate visits, and advocating for better food quality. Alicia said, “I was proud of him.” She admired his resourcefulness and advocacy within the prison. When Alicia’s family was informed after two long years that Arquímedes’ court hearing had at last been scheduled, Alicia told herself, “Finally, his innocence will be proven.”

## Politicizing Familial Love

Alicia was in for a shocking surprise. On December 20, 1996, the Salvadoran criminal court found Arquímedes guilty of murder, sentencing him to 25 years in prison. Despite the severity of the charges, his sentence was commuted to 13 years for serving years in prison without due process. Arquímedes’ conviction unraveled Alicia’s world. The prosecutor portrayed Arquímedes as a gang leader and claimed that the murder he had allegedly committed was related to his gang activity. Alicia rejected the prosecutor’s arguments. She knew that Arquímedes had been incarcerated without a trial for nearly two years. Because she believed in Arquímedes’ innocence, she saw the charges as “a lie…, a ploy by the prosecutor’s office because they couldn’t find anything to incriminate him, except that he belonged to a gang.” At the same time, the idea that her cousin was a gang member seemed inconceivable, a distant reality reserved for “others, not our family.” In recounting this moment, Alicia emphasized the family’s collective denial as they struggled to reconcile the state’s accusations with their perceptions of Arquímedes and themselves as a family. The family’s denial reflected the broadly held perception of gangs as inhuman and alien to their lives. Among Salvadoran families, including Alicia’s, gang membership was increasingly regarded as one of “the worst” outcomes for the person, their families, and society. This perception stemmed from the social stigma associated with gang involvement, violence, criminal activity, and economic disenfranchisement. Alicia was grappling to reconcile the new reality that she and her family confronted and the social implications for their family if her cousin’s membership in a gang were confirmed.

Alicia was upset. In seeking an explanation, she attributed Arquímedes’ troubles with the law to the influences encountered at his uncle’s mechanic’s shop. She cited Arquímedes’ exposure to alcohol and ex-military men’s lifestyles as the reasons for his drinking on the night of his arrest. At the same time, Alicia’s mother began a fact-finding mission to check the veracity of the prosecution’s claims regarding Arquímedes’ gang membership. Alicia recalled, “My mother asked my Uncle Ulises, ‘Did you know about it?’ He replied, ‘Yeah, he had bad company before he started coming to the shop’.” Alicia recalls her mother asking her cousin Mario the same question. “Mario replied, ‘Yes, and that’s why we used to fight.’ In her shock and dismay, Alicia realized, “Everyone knew he belonged to the gang except us!”

The confirmation of Arquímedes’ gang affiliation shattered Alicia’s perception of her family and its place in society. She likened the experience to a “bad dream” where denial becomes a defense mechanism against the painful truth. “You are seeing the evidence. It’s true, but you keep denying those possibilities. How could it be? Why so? Why not?” The stigma associated with gang membership, she explained, was pervasive, akin to having a dreaded disease like “leprosy” or “cancer.” This stigma further compounded Alicia’s struggle to accept Arquímedes’ gang membership and reinforced her family’s sense of isolation and shame. Yet even while she continued to deny Arquímedes’ gang membership, Alicia began to visit him regularly again, gaining insights into his perspective on incarceration. Alicia remembers that Arquímedes began expressing gratitude for his incarceration, believing it had provided him a window through which to understand the lives and struggles of others that he might not have otherwise learned. Arquímedes became involved with one non-profit organization focused on prisoner reintegration, and eventually co-founded another. With a gleam in her eyes, Alicia described how the cousin she had known resurfaced, demonstrating his caring for others.

Alicia witnessed how in each prison Arquímedes’ advocacy for the well-being of the incarcerated created conflict with prison guards and authorities. Alicia told me, “At Ciudad Barrios prison, authorities tightened security measures, conducting searches and confiscating illicit cellphones.” She later learned that these searches were an attempt by the prison authorities to dismantle not just gang members’ communication with the outside world but also Arquímedes’s authority as “prison lord.” This was a critical moment for Alicia. She was forced to finally accept that her beloved cousin was indeed “a gang member.” Even so Alicia interpreted his membership and leadership in the gang as an extension of his protector role.

Alicia’s perception of the gang evolved from viewing it as a malignant disease or “cancer” to rationalizing it as something potentially positive, specifically because of Arquímedes’ leadership role in the gang. “I thought, ‘Arquímedes is my cousin; he’s my family, and it’s okay… He always cared for and protected me. And he is also a boss, a gang leader who continues to guide and protect.” She expressed unwavering faith in his good judgment, emphasizing, “he is intelligent.” Her shift in perspective highlights the complex interplay of her love and care. It illustrates how the patriarchal notion of protection played a role in normalizing gang activity within her intimate familial relationship.

Alicia’s observation of the poor living conditions experienced by the incarcerated further cemented her belief in Arquímedes’ caring role as a human rights advocate. She stated, “I would visit him whenever I could, like once a month. Elizabeth, you know it is a long way to go from San Salvador. Hours of my life by bus to see him for 20 minutes.” According to Alicia, Arquímedes had zero privileges in prison. At one point, he shared a two-by-two-meter cell with 14 other prisoners. Another time, the authorities increased tension in the prison by placing a small group of MS-13 members in an isolated corridor surrounded by cells of rival *Barrio 18* gang members. Alicia’s observations of the overcrowded prison and the manipulation of gang rivalries by prison authorities as a means to maintain control furthered her determination to advocate for her cousin and his demands for just treatment of the incarcerated.

Alicia was about to graduate from law school when Arquímedes explained his worldview of the gangs. He told her, “We are humans.” Alicia told me that she replied in her head, “yes, they are human.” He went on to explain, “We [the gang] are a family.” Alicia disagreed, asserting, “I am your family.” She was upset and wanted to challenge Arquímedes’ understanding of ‘family’, but at that moment, Arquímedes asked her, “Can you help me? I want to write a letter, but my writing skills are rusty…” And so she did. For Alicia, this was a moment of tension and shared vulnerability that transcended the stigma of gang affiliation and prompted her to offer her help, writing a letter advocating to improve prison conditions. As Alicia described, it was at that point that “I became more involved in Arquímedes’ situation.”

## Navigating the Carceral Peace

Women relatives of gang members are often forced to spend months searching for their loved ones when they are arrested or transferred, generally relying on their social networks rather than the authorities to find them. One day, after an arduous bus journey, Alicia was met with the disorienting news that Arquímedes had been transferred to another prison. “He’s gone,” the prison guard told her, without providing further information about his new location. As Alicia started to leave, frustrated by this news, the mother of another incarcerated gang member came up to her and said, “Doggy asked me to tell you to come and see him.” Alicia obtained permission to see Doggy, who handed her a backpack filled with Arquímedes’ belongings and told her where he had been transferred. The abruptness and secrecy of the transfer underscores how prison authorities disregard the time and resources women put into visiting incarcerated relatives. Prison authorities place the burden of finding the location and dealing with the logistical hurdles of transporting their relatives’ belongings on prisoners’ female relatives. This added another layer to Alicia’s complex practice of solidarity.

During her first visit to Arquímedes’ new prison, Alicia recalled talking with him via speakerphone while separated by a glass wall. Alicia found her cousin in a deteriorating physical state: “When I saw Arquímedes, he had lost a significant amount of weight, 40 or 60 pounds. We only had 15 minutes to talk. I spent 10 minutes crying.” Alicia recounted asking Arquimedes, “What have they done to you? You are green. You are thin. Arquímedes replied, ‘Calm down so I can talk. What happens is that you come in here… [and] the guards beat you. And I am in an area that gets no sunlight’.” For Alicia, his significant weight loss, the visible bruises from beatings, and his story of being confined in a basement cell deprived of sunlight all testified to the dehumanizing conditions her cousin was enduring under the new punitive security policies in El Salvador.

Alicia recalled that the shift in punitive security policies also brought about stricter visitor regulations, requiring her to pre-register and provide identity photos and birth certificates. The heightened security measures included the incarcerated having to wear white uniforms that were to be provided by their relatives. Alicia recalled, “they asked for strapped white tennis shoes, white socks, white boxers, and white cotton T-shirts.” Alicia remembered going to downtown San Salvador, walking along rows and rows of shops full of white clothes. She said, “you went there, bought the items, and quickly put them in your backpack. You wanted to avoid people’s stigma. If they saw you buying white clothes, they knew you had a gang member relative in prison.” Having to buy the uniforms and find a way to carry them to the prisons added not only an economic burden to her family’s economy, but also the stress of having to conceal this reality from neighbors and other Salvadorans.

## Hope for the Future

In 2008, Arquímedes was released from prison. Alicia felt renewed hope for his future. Arquímedes lived with his brother in San Salvador and frequently visited Alicia on weekends. Alicia remembered, “we met every Sunday at my house. We played Scrabble and chess. We also talked about books. We maintained this dynamic for about six months.” Arquímedes then told her he wanted to “do something with his life.” He sought to enroll in philosophy courses at the national university, which seemed to signal a positive shift to Alicia. Her optimism quickly faded as Arquímedes resumed his active involvement with MS-13. She found his attitude towards violence deeply disturbing, once remarking that “life seems to be worth nothing to them.” One day, Mario came to Alicia's house, upset about Arquímedes’ violence towards a homeless man. The man would aggressively panhandle and masturbate in the alley next to Mario’s home. Arquímedes expressed concern that his 6-year-old niece would be exposed to that behavior and asked the man to stop bothering people. But the behavior continued. After several warnings, Arquímedes beat the man. When Alicia heard about this, she confronted Arquímedes, who dismissed her concerns, saying, “he was just a homeless man, a nobody. I don’t see what your drama is. Why are you questioning me?” Alicia remembered that Arquímedes was unmoved, justifying his actions as necessary to protect his family and community.

Their conflicting views on morality and violence led to increasingly tense disagreements, which persisted even when Arquímedes subsequently participated in the negotiation of the abovementioned gang truce. When he told her about his participation, she responded “why are you telling me this? What do you think? That I am going to protect you? You continue to do bad things.” Arquímedes curtly told her, “we must take care of ourselves,” but nevertheless promised Alicia that he would retire from the gang. Despite their differences and increasingly tense relationship, Alicia was optimistic that this might happen, even though the truce process came to an end in 2014.

Her hopes were dashed, however, when Arquímedes was arrested in mid-2016 under new anti-terrorism laws. These laws created a distinct criminal process, and Alicia was able to attend the specialized court where the pre-trial charges were read and learn where Arquímedes was being held. The following day, she visited him, presenting herself as his lawyer to secure an interview with him. Alicia explained that this unconventional approach caused confusion among the prison guards, who lacked a protocol for such visits: “The novelty of a lawyer visiting a client highlighted the unusual nature of my request and the lack of established procedures for legal representation within the prison. I brought Arquímedes essential supplies – clothing, toothbrush, toothpaste, soap, and shampoo – all carefully placed in a transparent bag and labeled with his name,” adhering to the strict regulations governing personal items. The conditions Arquímedes endured were appalling. He was confined to “a cramped, humid cell, barely two by ten meters, crammed with 150 other inmates,” conditions that created a breeding ground for disease.

Securing further visits proved to be an ordeal, however, fraught with obstacles created by state officials and state surveillance. “In the prison, the guards would ask me: ‘Why do you have the same last name?’ They said that the photo on the lawyer’s ID card didn’t look like me.” They contacted the Supreme Court of Justice for verification, and then said, “we cannot let you pass because the Court isn’t responding.” Alicia also told me that during this period, she thinks she was followed by government agents. In court, the prosecutor accused her of collaborating with the gang, although the judge ultimately dismissed the charges against her.

Alicia managed to visit with Arquímedes only once more during the pre-trial period. She said Arquímedes was persuaded that the charges against him were politically motivated, stemming from his involvement in the gang truce and his testimony before the Inter-American Court of Human Rights. He believed he was being targeted for speaking out about missing persons. Hearing Arquímedes reinforced Alicia’s belief that her cousin had embraced a sense of care that drove him to engage in peace negotiations with the state and advocate for the rights of the incarcerated and criminalized. It drove her to continue supporting his case.

A subsequent cancer diagnosis forced Alicia to give up her position as Arquímedes’ lawyer. At the same time, the Salvadoran government stepped up its anti-gang rhetoric. It accused a former British ambassador of supporting gang members and forced him to leave the country. Feeling vulnerable to the political backlash, the Salvadoran NGOs that had offered legal support to Arquímedes withdrew it. In the absence of proper representation, he was sentenced to 41 years in prison for extortion and terrorism. A profound sense of grief consumed Alicia. “I cried. I thought: ‘When he had just started to do things right…’.” Alicia remembered her sense of loss for the life he could no longer live, to “enjoy his children and watch them grow.” Witnessing Arquímedes’ subsequent physical and emotional deterioration in prison over the next few months was heartbreaking: “I saw him become half of what he had been. He was very thin…”.

Although Alicia knows that Arquímedes committed violent acts during his years with the gang that she cannot condone, she fundamentally rejects the system that shows him “zero rights, zero humanity” and that only wants “to kill him, to silence him.” She still cares for him, and feels a lingering sense of responsibility, questioning whether she could have done more, while recognizing the limits of her influence, love, and concern for him. She is still coming to terms with a complex mix of emotions that underscore the enduring bonds of family and the challenges of reconciling love with accountability. Nevertheless, she told me, “I now see with whatever objectivity I can have that I was always clear with him. There’s no way you say things metaphorically when you speak out of love and understanding.”

## Women’s Complex Politics of Solidarity

Alicia’s story highlights the essential role that women play in contributing to the economic and emotional support of their incarcerated gang member relatives. It demonstrates their tireless efforts to ensure their loved ones’ basic needs are met, as well as the solidarity they share with one another. Alicia’s intimate understanding of “family” as including gang members is key to challenging the dominant representation of gangs as purely violent, criminal entities. The efforts of gang members and their families to support one another reveals their strong sense of community and mutual aid, highlighting the importance of the networks they’ve created in order to navigate the challenges of incarceration and the prison system. Many Salvadoran women in the post-Accords era have found themselves in a similar position to Alicia’s, negotiating notions of protection, family, love, and the state’s characterization of their relatives as an internal enemy and a “plague.” This complex struggle has often transformed women’s life trajectories.

Women’s complex politics of solidarity, embodied by Alicia’s story, lays bare the particular construction of El Salvador’s carceral peace. Salvadoran women’s politics of solidarity assert their agency and challenge the binary and Manichean understanding of carceral peace, which rests on a patriarchal notion of protection that imagines an internal enemy and criminal figure against which the (masculinist) state must fight to ensure the vulnerable (women and children) who make up the majority of the population can live and prosper. They are push us to think beyond dominant imaginaries and moral conceptions to envision humanity in a broader relational manner, one that implies a different notion of community, peace, and living.

At the same time, women’s Politics of solidarity in El Salvador are largely rooted in their understanding of family bonds. Salvadoran culture places great importance on family relationships and supporting each other during difficult times, which is one reason why the moral underpinnings of carceral peace are framed in familial terms. Women go to great lengths to accompany and support their relatives as they move through the prison system. Initially, Alicia’s advocacy for Arquímedes was grounded in her conviction that he was innocent. But after the court found Arquimedes guilty, and as she sought to know more about his life in the gang, she developed new understandings both of her cousin’s actions and the repressive role of the Salvadoran state. While she continued to be a caretaker, bringing food and attempting to soften the harshness of his prison sentence, she also advocated to end Arquímedes’ incarceration, and became the key supporter of his fight against human rights violations in the prison. Her love and care for him as a family member led her to develop a broader consciousness that, in turn, led her to advocacy work.

Salvadoran women’s Politics of solidarity are complex, ranging from caring for their relatives inside and outside of prison to sharply critiquing their violence. While women’s role as family caretakers is generally known, their role as caretakers of their communities – and, indeed, their societies – is less acknowledged. Many women in El Salvador are militant in challenging the practices of violence of their male relatives in gangs while at the same time advocating for their rights as human beings and criticizing the patriarchal repression of the state. They frequently do this through multi-layered care work not only in opposition to the state, but also with limited support from not-for-profit organizations and extended family. Most of the time, this care work is clandestine. The complex burdens placed on women result in frustration, depression, anxiety, exhaustion, illnesses, and push-pull dynamics in their relationships with their male relatives. Yet they continue to engage in these complex practices of solidarity that stem from their love, their desire that their relatives attain a better life outside the gang, and their urgent critique of a state and society that denies their families this opportunity.

## Footnotes

1 See: https://www.graduateinstitute.ch/research-centres/centre-conflict-development-peacebuilding/gangs-gangsters-and-ganglands-towards.

2 I have used pseudonyms throughout the manuscript at the request of Alicia.

3 Unlike some countries, one must first be a lawyer before they can be a notary in El Salvador,

4 “Quezalte” is an abbreviated name for Quezaltepeque prison.

## References

[CR1] Abu-Lughod, L. (2002). Do Muslim Women Really Need Saving? Anthropological Reflections on Cultural Relativism and Its Others. *American Anthropologist*, 104(3): 783–790.

[CR2] Amar, P. (2013). *The Security Archipelago: Human-security States, Sexuality Politics, and the End of Neoliberalism*. Duke University Press.

[CR3] Amnesty-International. (2024a). El Salvador, A Thousand Days into the State of Emergency: “Security” at the Expense of Human Rights. https://www.amnesty.org/en/latest/news/2024/12/el-salvador-mil-dias-regimen-excepcion-modelo-seguridad-a-costa-derechos-humanos/.

[CR4] Amnesty-International. (2024b). El Salvador: The Institutionalization of Human Rights Violations after Two Years of Emergency Rule. https://www.amnesty.org/en/latest/news/2024/03/el-salvador-two-years-emergency-rule/.

[CR5] Aretxaga, B. A. (1997). *Shattering Silence: Women, Nationalism, and Political Subjectivity in Northern Ireland*. Princeton University Press.

[CR6] Ayala, E. (2013). *Impunity, Machismo Fuels Femicides in El Salvador*. Inter Press Service, April 10, http://www.ipsnews.net/2013/04/impunity-machismo-fuel-femicides-in-el-salvador/.

[CR7] Baird, A. (2021). “Man a Kill a Man for Nutin”: Gang Transnationalism, Masculinities, and Violence in Belize City. *Men and Masculinities*, 24(3): 411–431.

[CR8] Brotherton, D. C. (2015). *Youth Street Gangs: A Critical Appraisal*. Routledge.

[CR9] Bruneau, T. C., Dammert, L. a., & Skinner, E. (2011). *Maras: Gang Violence and Security in Central America* (1st ed.). University of Texas Press.

[CR10] Collins, P. H. (2019). *Intersectionality as Critical Social Theory*. Duke University Press.

[CR11] Cosgrove, S. (1999). *Give them the Credit they Deserve: Marketwomen and the Impact of Micro-enterprise Lending in the Municipalities of Apopa and Nejapa, El Salvador*. Unpubished PhD Dissertation, Northeastern University, Boston.

[CR12] Crenshaw, K. (2012). From Private Violence to Mass Incarceration: Thinking Intersectionally about Women, Race, and Social Control. *UCLA Law Review*, 141, https://scholarship.law.columbia.edu/faculty_scholarship/2863.

[CR13] Cruz, J. M. (2014). La Transformación de las Maras Centroamericanas. *Cuestiones De Sociología*, 10, https://www.cuestionessociologia.fahce.unlp.edu.ar/article/view/CSn10a14.

[CR14] Das, V. (2007). *Life and Words: Violence and the Descent into the Ordinary*. Berkeley: University of California Press.

[CR15] Gilmore, R. W. (2022). You Have Dislodged a Boulder: Mothers and Prisoners in the Post-Keynesian California Landscape. In B. Bhandar & A. Toscano (Eds.), *Abolition Geography: Essays Towards Liberation*. London: Verso.

[CR16] Grimaldi Calderon, G. (2024). *Carceral Intimacies: Family, Racialization, and El Salvador’s Carceral State under Authoritarian Politics*. Unpublished PhD Dissertation, University of Illinois at Urbana-Champaign, Urbana.

[CR17] Hagedorn, J. M. (2008). *A World of Gangs: Armed Young Men and Gangsta Culture*. Minneapolis: University of Minnesota Press.

[CR18] Hallsworth, S., & Young, T. (2008). Gang Talk and Gang Talkers: A Critique. *Crime, Media, Culture*, 4(2): 175–195.

[CR19] Hill Collins, P. (2019). *Intersectionality as Critical Social Theory*. Durham: Duke University Press.

[CR20] Hinton, E. K. (2016). *From the War on Poverty to the War on Crime: The Making of Mass Incarceration in America*. Cambridge: Harvard University Press.

[CR21] Hirschi, T. (2002). *Causes of Delinquency*. New York: Routledge.

[CR22] Hume, M. (2007). Mano Dura: El Salvador Responds to Gangs. *Development in Practice*, 17(6): 739–751.

[CR23] Hume, M. (2008). The Myths of Violence: Gender, Conflict, and Community in El Salvador. *Latin American Perspectives*, 35(5): 59–76.

[CR24] Hume, M. (2009). *The Politics of Violence: Gender, Conflict and Community in El Salvador*. Chichester: Wiley-Blackwell.

[CR25] IUDOP (Instituto Universitario de Opinión Pública). (2010) *“Segundos en el aire”: Mujeres Pandilleras y sus Prisiones*. San Salvador: Talleres Gráficos UCA.

[CR26] Jensen, S., & Rodgers, D. (2022). The Intimacies of Drug Dealing: Narcotics, Kinship and Embeddedness in Nicaragua and South Africa. *Third World Quarterly*, 43(11): 2618–2636.

[CR27] Kontos, L., Brotherton, D., & Barrios, L. (Eds.). (2003). *Gangs and Society: Alternative Perspectives*. New York: Columbia University Press.

[CR28] Melossi, D. (2008). *Controlling Crime, Controlling Society: Thinking About Crime in Europe and America*. Cambridge: Polity.

[CR29] Mohanty, C. T. (1984). Under Western Eyes: Feminist Scholarship and Colonial Discourses. *Boundary 2*, 12/13: 333–358.

[CR30] Moodie, E. (2010). *El Salvador in the Aftermath of Peace: Crime, Uncertainty, and the Transition to Democracy*. Philadelphia: University of Pennsylvania Press.

[CR31] Naber, N., Naser, S., & Strong, J. (2020). Radical Mothering for Abolitionist Futures Post-Covid-19. *Abolitionjournal.org*. https://abolitionjournal.org/radical-mothering/.

[CR32] Nowak, M. (2012). Femicide: A Global Problem. *Small Arms Survey Research Notes: Armed Violence*, no. 14, http://www.smallarmssurvey.org/fileadmin/docs/H-Research_Notes/SAS-Research-Note-14.pdf.

[CR33] Pyrooz, D. C., Densley, J. A., & Leverso, J. (Eds.). (2024). *The Oxford Handbook of Gangs and Society*. Oxford: Oxford University Press.

[CR34] Rodgers, D. (2024). Soraya, la Reina del Sur in Nicaragua. In J. Auyero (Ed.), *Portraits of Persistence: Inequality and Hope in Latin America*. Austin: University of Texas Press.

[CR35] Shirlow, P., & Dowler, L. (2010). ‘We Women No More’: Female Partners of Republican Political Prisoners in Belfast. *Environment and Planning A: Economy and Space*, 42(2): 384–399.

[CR36] Silber, I. C. (2011). *Everyday Revolutionaries: Gender, Violence, and Disillusionment in Postwar El Salvador*. New Brunswick: Rutgers University Press.

[CR37] Stephen, L. (1997). *Women and Social Movements in Latin America: Power from Below*. Austin: University of Texas Press.

[CR38] Ticktin, M. I. (2011). *Casualties of Care: Immigration and the Politics of Humanitarianism in France*. Berkeley: University of California Press.

[CR39] Velásquez Estrada, R. E. (2022). Intersectional Justice Denied: Racist Warring Masculinity, Negative Peace, and Violence in Post-peace Accords El Salvador. *American Anthropologist*, 124(1): 39–52.

[CR40] Velásquez Estrada, R. E. (2026). Fugitive Collaborative Research: Seeking Mutuality as Research Accountability. In S. Cordis, M. J. Berry, C. Chávez Argüellez, S. Ihmound, & R. E. Velásquez Estrada (Eds.), *Fugitive Anthropology: Embodying Activist Research*. Austin: University of Texas Press, forthcoming.

[CR41] Viterna, J. (2013). *Women in War: The Micro-processes of Mobilization in El Salvador*. Oxford: Oxford University Press.

[CR42] Watts, J. (2015). One Murder Every Hour: How El Salvador Became the Homicide Capital of the World. *The Guardian*, 22 August, https://www.theguardian.com/world/2015/aug/22/el-salvador-worlds-most-homicidal-place.

[CR43] Wolf, S. (2017). *Mano Dura: The Politics of Gang Control in El Salvador*. Austin: University of Texas Press.

[CR44] Young, Iris M. (2003). The Logic of Masculinist Protection: Reflections on the Current Security State. *Signs: Journal of Women in Culture and Society*, 29(1): 1–25.

[CR45] Zilberg, E. (2011). *Space of Detention: The Making of a Transnational Gang Crisis Between Los Angeles and San Salvador*. Durham: Duke University Press.

